# Chopstick operation training with the left non-dominant hand

**DOI:** 10.1515/tnsci-2020-0189

**Published:** 2021-10-22

**Authors:** Daisuke Sawamura, Satoshi Sakuraba, Kazuki Yoshida, Naoya Hasegawa, Yumi Suzuki, Susumu Yoshida, Toshihiro Honke, Shinya Sakai

**Affiliations:** Department of Rehabilitation Science, Faculty of Health Sciences, Hokkaido University, Sapporo, Hokkaido, 060-0812, Japan; Department of Rehabilitation Sciences, Health Sciences University of Hokkaido, Ishikari-Gun, 061-0293, Japan; Department of Occupational Therapy, Yamagata Prefectural University of Health Sciences, Yamagata, 990-2212, Japan

**Keywords:** motor skill acquisition, asymmetry, tool use, movement smoothness, fNIRS

## Abstract

**Background:**

Training a non-dominant hand is important for rehabilitating people who are required to change handedness. However, improving the dexterity in using chopsticks with a non-dominant hand through training remains unclear. This study is aimed to measure whether chopstick training improves non-dominant hand chopstick operation skills and leads to acquisition of skill levels similar to those of the dominant hand.

**Methods:**

This single-blinded randomized controlled trial enrolled 34 healthy young right-handed subjects who scored >70 points on the Edinburgh Handedness Questionnaire Inventory. They were randomly allocated to training or control groups. The training group participated in a 6-week chopstick training program with the non-dominant left hand, while the control group did not. Asymmetry of chopstick operation skill, perceived psychological stress, and oxygen-hemoglobin concentration as a brain activity measure in each hemisphere were measured before and after training.

**Results:**

Participants in the training group had significantly lower asymmetry than those in the control group during the post-training assessment (*F*[1,30] ≥ 5.54, *p* ≤ 0.03, partial *η* ^*2*^ ≥ 0.156). Only perceived psychological stress had a significantly higher asymmetry during the post-training assessment (*t*[15] = 3.81, *p* < 0.01).

**Conclusion:**

Six weeks of chopstick training improved non-dominant chopstick operation skills, and a performance level similar to that of the dominant hand was acquired.

## 1 Introduction

The utilization of chopsticks requires handedness-specific and complex and fine motor skills. Approximately 70–80% of stroke patients have upper extremity paralysis, which compromises the motor skills necessary for activities of daily living [1,2]. Only 20% of them completely recovered [1]; therefore, they were required to change handedness. However, the mechanism and neural activity underlying training of the non-dominant hand and its effect on the acquisition of motor skill remains unclear.

The dominant hand has better performance speed of manual dexterity [3,4,5], accuracy, and variability of reach movements than the non-dominant hand [6,7]. These motor functions enable the skillful use of chopsticks. Furthermore, it has been reported that long-term repetitive training is necessary to improve these motor functions and the tool-use skills based on these motor functions [8]. Long-term repetitive training may then be needed for the non-dominant hand so that it is able to reach a performance level similar to that of the dominant hand.

Several studies have shown the effectiveness of tool-use training of the non-dominant hand [9,10,11]. Philip and Frey [9] reported that precision drawing using the non-dominant hand for 30 min every day for 10 days significantly improved the smoothness of movement and speed of the non-dominant hand, which significantly narrowed the difference in performance between the two hands. Sandve et al. [10] reported that non-dominant handwriting significantly improved and closely matched that of the dominant hand after 15 days of training. Schweiger et al. [11] reported that computer mouse training with the non-dominant hand for 15 min every day, 5 days per week, for 6 weeks significantly improved the hand and closely matched the performance of the dominant hand. Skill proficiency is highly dependent on the nature of the task. Moreover, effort is needed to acquire complex and fine motor skills [8]. The training time and extent to which skills can be acquired – compared to those of the dominant hand – within a given period provide valuable information that can be used in the rehabilitation of people who need to change their handedness. We measured the training effect of chopstick utilization with the non-dominant hand and focused on training-derived changes in brain activity [12].

An important method for supporting the assessment of skill acquisition is to evaluate perceived psychological stress which, if experienced during task performance, can impair efforts to be physically active and motivated [13,14]. Stress interferes with one’s engagement with activity [14,15]. In addition, we measured brain activity to understand the mechanism of action and neural activity underlying the non-dominant hand’s acquisition of skill and the effect of training on the acquisition of this skill. Systematic reviews and meta-analyses [16,17,18,19,20] identified brain regions related to motor skills, which included the dorsolateral prefrontal cortex (DLPFC), premotor cortex (PMC), and primary sensory motor (SM) cortex. These regions facilitated motor learning. Their activity gradually increased or decreased throughout the motor learning process. Previous studies have shown that specific brain activity patterns in the left hemisphere are associated with tool use [21,22,23] and the dynamics of limb trajectory [24,25], independent of handedness.

We have previously reported on training-derived changes in brain activity using the non-dominant left hand [12]. The study presented here is aimed to determine comprehensively whether this training can improve the non-dominant chopstick operation skills sufficiently and lead to the acquisition of a performance level similar to that of the dominant hand within the timeframe of a general period of rehabilitation. This study thus focused on training-derived changes in left-right asymmetries of chopstick operation skills and perceived psychological stress, using the data on right-hand chopstick operation skills, perceived psychological stress, and brain activity in each hemisphere, which differed from our previous study.

## 2 Methods

### 2.1 Trial design, randomization, and blinding

This study was a single-blinded randomized controlled trial with stratification. Participants who met the inclusion criteria were randomly allocated to either the training or the control group by a researcher who was neither an evaluator nor a trainer. The training group received 6 weeks of training with the non-dominant hand with chopsticks for 30 min every day, 5 days per week. The control group did not receive any training.

The study was registered at clinicaltrials.gov, trial registration (Registered number: UMIN000044007).

### 2.2 Training procedure

The training group participants used their left non-dominant hand to repeatedly transfer various objects (e.g., marbles, beans, and sponges) of different sizes, weights, shapes, and materials from one dish to another with 21-cm long wooden chopsticks. All participants in the training group practiced operating the chopsticks continuously at our laboratory for 30 min a day under the surveillance of the trainers.

**Figure 1:**
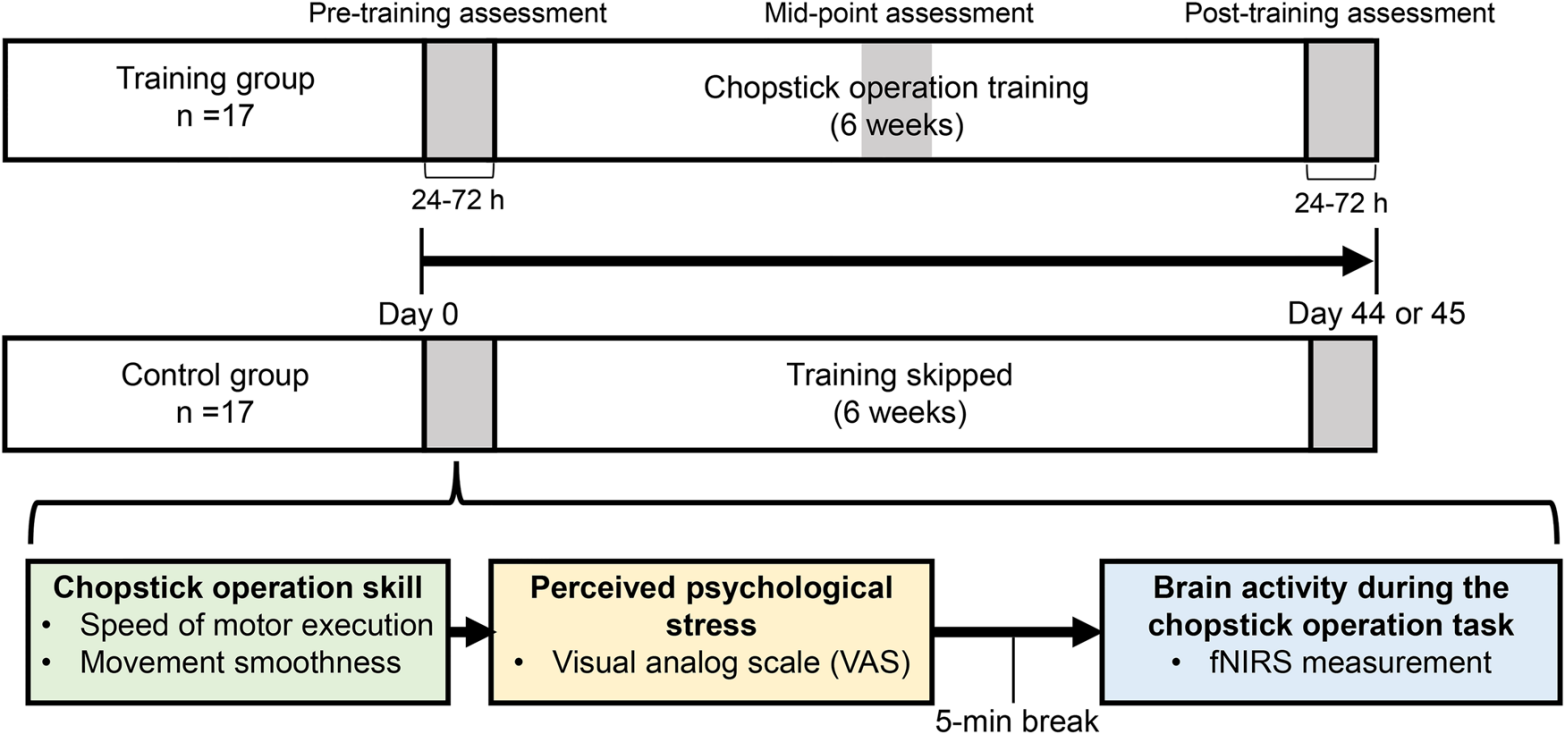
The experimental procedure followed in this study evaluated chopstick operation skill, perceived psychological stress, and brain activity three times (pre-, mid-, and post-training) for the training group and twice (pre- and post-training) for the control group. The mid-training assessment was excluded from the analysis as it did not form part of the purpose of this study. The post-training assessments were conducted 24–72 h after training to evaluate retention. fNIRS: functional near-infrared spectroscopy.

The experimental procedure followed in this study is illustrated in Figure 1. Chopstick operation skills with both hands and measurement of brain activity during the chopstick operation task were evaluated for the training group at three-time points: pre-training, after 3 weeks of training (training mid-point), and post-training. Evaluations for the control group were only conducted at two-time points: pre-training and post-training. The post-training assessments were conducted 24–72 h after the completion of training to evaluate the retention of chopstick operation skills. The mid-point assessment was excluded from the analysis because it did not form part of the outcomes of this study.

The smoothness of upper extremity movement, perceived psychological stress, and brain activity were evaluated during the marble transferring task. The details of the experimental procedure, chopstick operation task, and functional near-infrared spectroscopy (fNIRS) are described in our previous study [12].

### 2.3 Participants and sample size

The required sample size for this study was determined by a priori power analysis using G*Power 3.1.12 (Heinrich Heine University, Düsseldorf, Germany). Regarding input parameters, the effect size (Cohen’s *d* = 1.09) was calculated based on a previous study that performed chopstick operation training with the non-dominant hand [26]. The sample size estimate for training group comparison based on significance probability (*α* = 0.05), statistical power (1 − *β* = 0.95), and effect size (*d* = 1.09) resulted in *n* = 13. To ensure a conservative estimation, we added 20–30% to account for possible dropout and outliers, and we finally planned for a sample size of *n* = 17. To make the analysis of the training effect more robust, the control group was also set to *n* = 17.

Thirty-four healthy right-handed volunteers were recruited from September 2016 through August 2017, according to the following inclusion and exclusion criteria. Inclusion criteria were: (1) age range of 20–35 years, (2) achieving over 70 points on the Edinburgh Handedness Questionnaire Inventory [27], and (3) no experience using chopsticks with their left non-dominant hand on a daily basis. Exclusion criteria were: (1) history of neurological or psychiatric disorders and (2) functional limitations of both or either upper limbs that might affect use of chopsticks. The participants selected for this study were the same ones who participated in previous studies [12]. One subject from the training group failed to complete the training, and one from the control group was unable to accomplish the re-assessment. Thus, 16 participants each were included in the training (mean age, 21.1 ± 0.7 years; 8 women) and control (mean age, 21.3 ± 0.8 years; 8 women) groups.


**Informed consent:** Informed consent has been obtained from all individuals included in this study.
**Ethical approval:** The research related to human use has been complied with all the relevant national regulations, institutional policies, and in accordance with the tenets of the Helsinki Declaration, and has been approved by the authors’ institutional review board or equivalent committee (approval number: 16R033032).

### 2.4 Outcome measures

#### 2.4.1 Speed of motor execution

The execution speed was the elapsed time in transferring an object from one dish to another using a pair of 21 cm wooden chopsticks. Two round dishes (diameter, 13 cm; height, 5 cm) were placed 20 cm to the left and right of the participants’ midline. Participants sat on a chair and held the chopsticks in a vertical orientation 15 cm anterior to their midlines. This was the starting position. The participants transferred marbles from left to right with their left non-dominant hands, and from right to left with their right hands. The task was performed three times and the averaged execution time across trials was calculated.

#### 2.4.2 Movement smoothness

We measured the smoothness of the bilateral upper extremity joint movement during the utilization of chopsticks using a three-dimensional motion analysis device (myoMOTION™, Noraxon, USA). The motion sensors were placed over three body segments (upper thoracic below C7, upper arm, and forearm). The calculation of each joint angle was in accordance with the International Society of Biomechanics [28]. The measured target’s upper extremity joint movements included bilateral shoulder adduction-abduction, bilateral shoulder flexion-extension, and bilateral elbow flexion-extension. We calculated the angular jerk, which was the time derivative of the angular acceleration of a given joint, to represent smoothness. The root mean square jerk (RMSJ) was calculated as follows:

\text{RMSJ}=\sqrt{\frac{1}{N}\mathop{\sum }\limits_{i=1}^{N}J{(i)}^{2}},]

where *J*(*i*) is the jerk of the *i*th data point, and *N* is the total number of data points sampled from a given participant.

### 2.5 Perceived psychological stress

We measured the perceived psychological stress in the marble transferring task using a visual analog scale (VAS), which comprised a small, unmarked 100 mm horizontal line with endpoints labeled “no stress” and “worth imaginable stress.” Each participant provided a VAS score after performing the task.

### 2.6 fNIRS instrument

**Figure 2:**
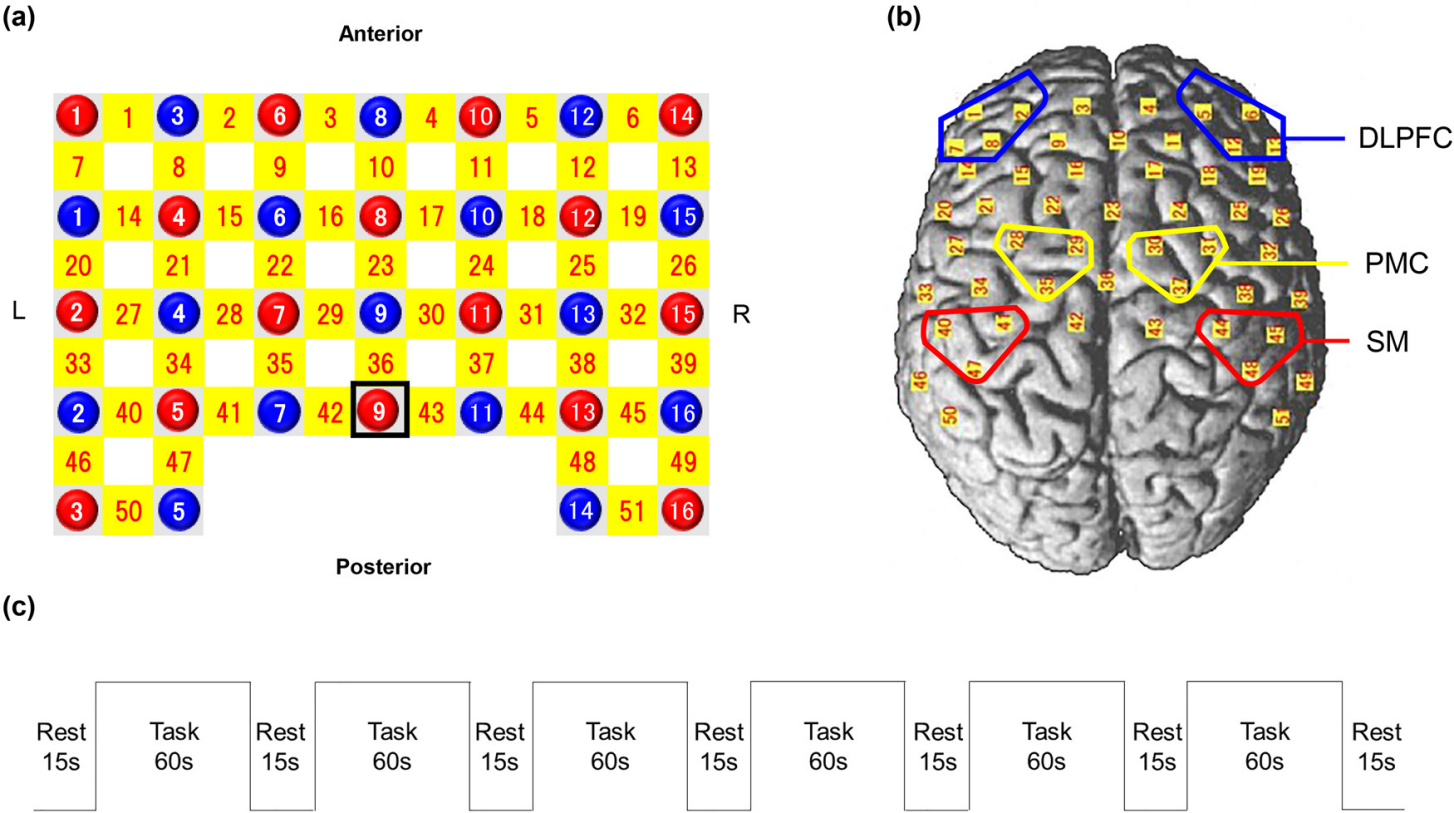
Channel configuration of the near-infrared spectroscopy probe set. (a) Red and blue circles show illuminators and detectors, respectively; yellow square shows near-infrared spectroscopy channels. Illuminator 9 was shown as Cz according to the international 10/20 placement system. (b) All channels are placed on the cortical surface. Blue, yellow, and red frames show the DLPFC, PMC, and SM cortex, respectively. (c) The experimental design of fNIRS measurements.

The changes in oxygen-hemoglobin (Oxy-Hb) concentration were measured using a fNIRS optical topography system (LABNIRS, Shimadzu. Kyoto, Japan) with three wavelengths of near-infrared light (780, 805, and 830 nm). Figure 2 shows the positions of the probe and channel. The probes were set over the DLPFC, PMC, and SM. The anatomical location of each channel was determined according to the Talairach Daemon database [29,30]. The changes in Oxy-Hb concentration were recorded since they indicate representative brain activity [31,32]. The block design comprised six task blocks, with three blocks in each hand. The sequences of the right- or left-hand tasks were randomly assigned (Figure 2). The six regions of interest (ROIs), three in each hemisphere, included the bilateral DLPFC (left channels: 2, 7, 8, and 14; right channels: 5, 12, 13, and 19), the bilateral PMC (left channels: 28, 29, and 35; right channels: 30, 31, and 37), and bilateral primary SM cortex (left channels: 40, 41, and 47; right channels: 44, 45, and 48). Task-related changes in Oxy-Hb concentrations in every channel in each hemispheric ROI were averaged over the time period of the task (0–60 s after task onset). Finally, the task-related Oxy-Hb concentration changes in each hemisphere were calculated by averaging the Oxy-Hb concentration changes in all channels in each hemisphere’s ROI.

### 2.7 Statistical analyses

The left-right asymmetry of all outcome values except for Oxy-Hb concentration in each hemisphere was calculated by subtracting the value in the right-hand chopstick performance from the value in the left-hand chopstick performance. The lower the asymmetry, the closer the left is to the right chopstick performance. The chopstick asymmetry of motion speed, smoothness, and perceived psychological stress were analyzed using a 2 × 2 mixed-design analysis of variance (ANOVA) with a group (training or control) as the between-subjects factor and time (pre-training or post-training) as the within-subject factor. In addition, planned contrasts were performed for the null hypothesis that the mean of the asymmetry in the training group was zero during post-training assessment. We did not use asymmetry in assessing the changes in Oxy-Hb concentration because the specific brain activity patterns in the left hemisphere were independent of tool-use handedness [21,22,23]. The changes in Oxy-Hb concentration in each hemisphere were analyzed using a 2 × 2 × 2 mixed-design ANOVA with the group as the between-subjects factor, and time and chopstick use (left- or right-hand chopstick use) as the within-subject factors.

We performed a correlation analysis of the changes in asymmetry of chopstick performance and perceived psychological stress, and changes in Oxy-Hb concentration in each hemisphere in the training group using Pearson’s product-moment correlation coefficient. Statistical analyses were performed using the SPSS software (version 25.0; IBM Corp., Armonk, NY, USA). Statistical significance was set at 0.05.

## 3 Results

### 3.1 Demographics

There were no significant differences in age, sex, and Handedness Questionnaire Inventory score between the training and control groups (age: *t*[30] = −0.47, *p* = 0.64; sex: *χ* ^2^[1, *n* = 32] = 0.00, *p* = 1.00; Handedness Questionnaire Inventory score: *t*[30] = 0.001, *p* = 0.99).

### 3.2 Speed of motor execution

**Figure 3:**
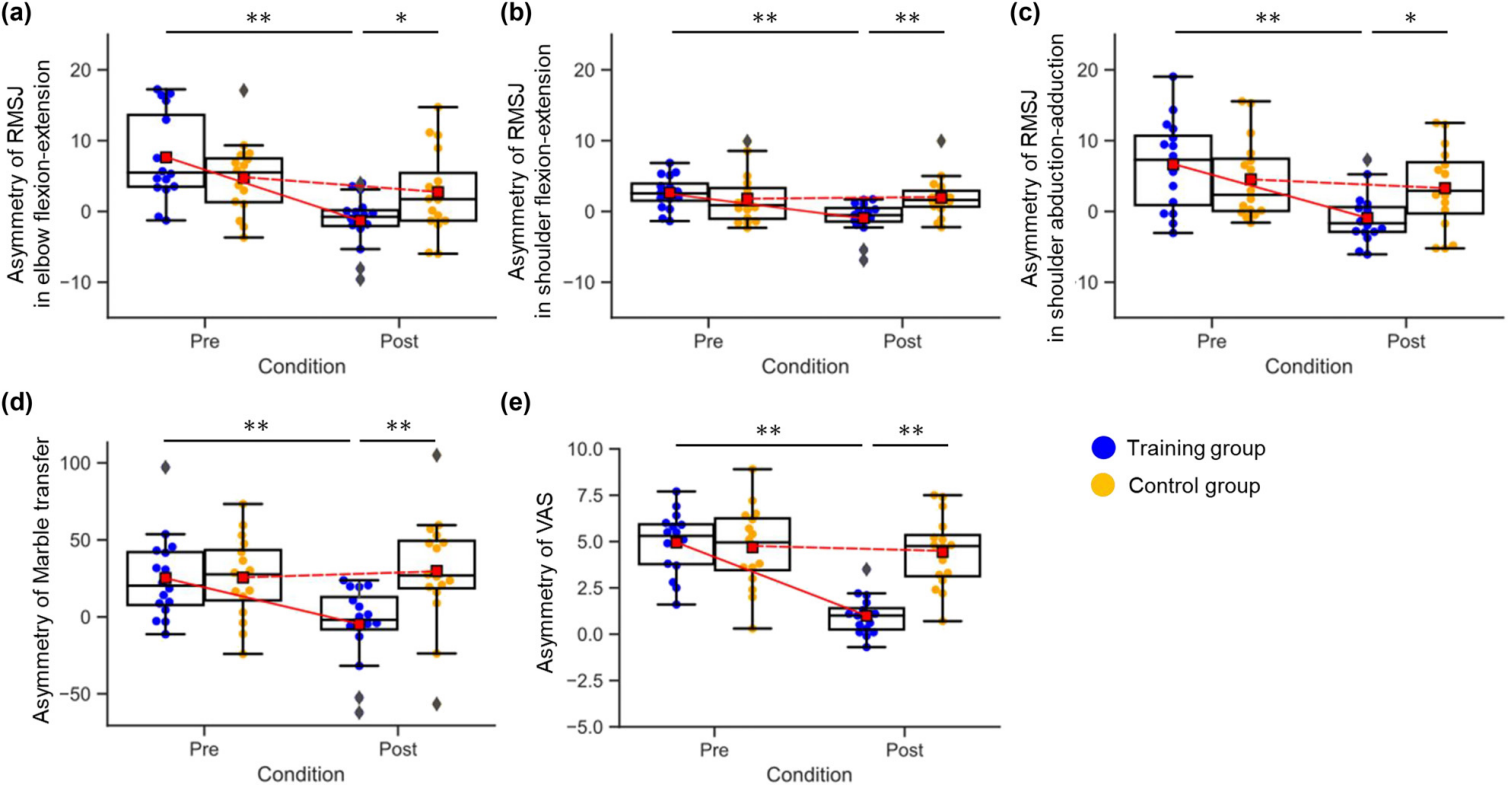
Significant training effect on chopstick operation and perceived psychological stress. (a) Asymmetry of the RMSJ in elbow flexion-extension. (b) Asymmetry of the RMSJ in shoulder flexion-extension. (c) Asymmetry of the RMSJ in shoulder abduction-adduction. (d) Asymmetry of the RMSJ in the completion time of object transfer. (e) Asymmetry of the VAS. Error bars indicate 95% confidence interval, the bottom and top of each box indicate the 25th and 75th percentiles, and the line and square inside the box indicate the 50th percentile (median) and the mean, respectively. Any outliers are shown as diamonds. * and ** refer to *p* < 0.05 and *p* < 0.01, respectively.

**Figure 4:**
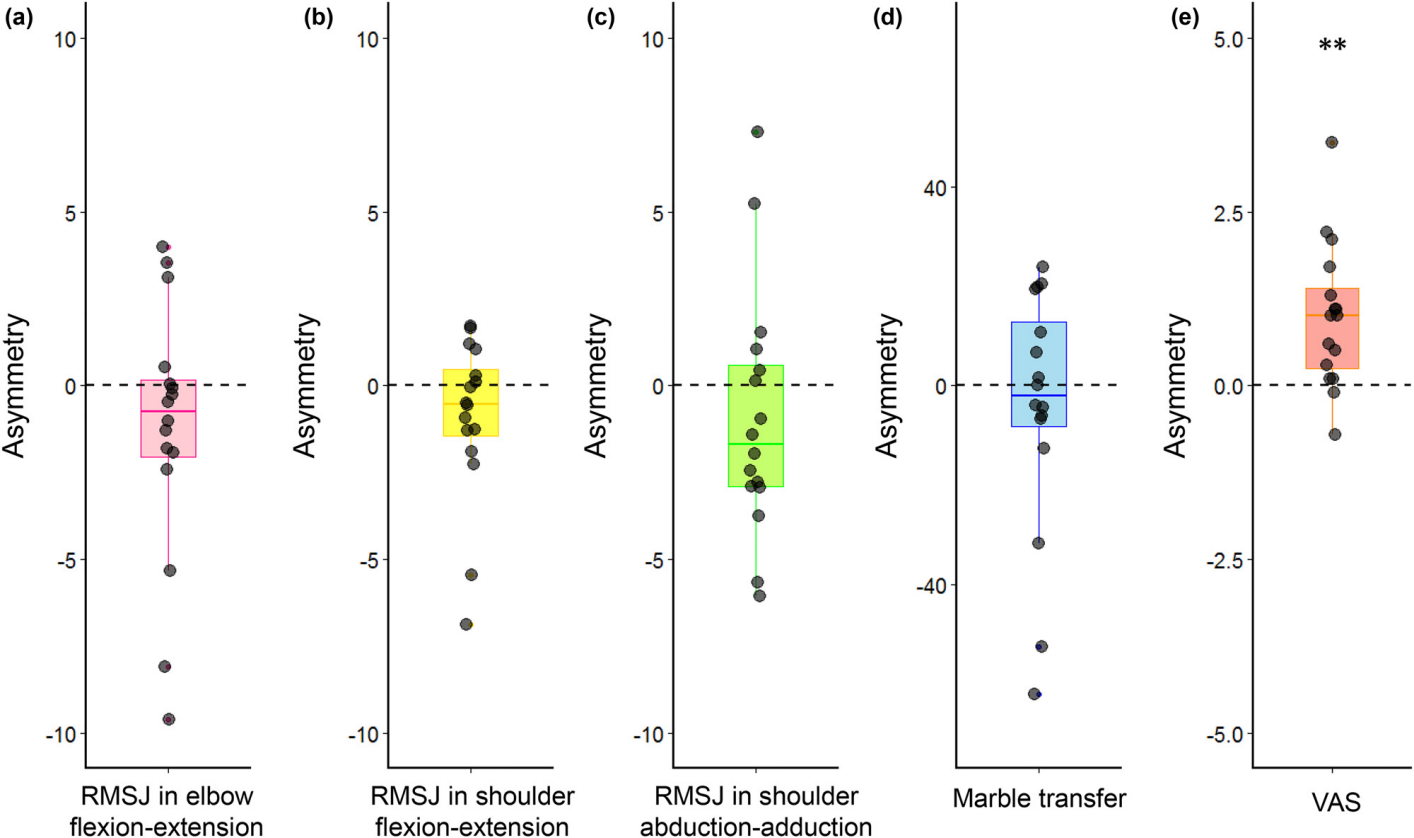
No significant difference in all chopstick operation measures except for the perceived psychological stress. (a) Asymmetry of the RMSJ in elbow flexion-extension. (b) Asymmetry of the RMSJ in shoulder flexion-extension. (c) Asymmetry of the RMSJ in shoulder abduction-adduction. (d) Asymmetry of the RMSJ in the completion time of object transfer. (e) Asymmetry of the VAS. The displayed points show the individual asymmetries. Error bars indicate 95% confidence interval, the bottom and top of each box indicate the 25th and 75th percentiles, and the line inside the box indicates the 50th percentile (median). ** refers to *p* < 0.01.

A 2 × 2 mixed-design ANOVA of the asymmetry in marble transfer time, with time (pre-training vs post-training) as the within-subject factor and group (training group vs control group) as the between-subjects factor, revealed a significant main effect of group (*F*[1,30] = 5.78, *p* = 0.02, partial *η* ^2^ = 0.162) and a significant time × group interaction (*F*[1,30] = 5.54, *p* = 0.03, partial *η* ^*2*^ = 0.156) (Figure 3). A *post-hoc t*-test showed that the asymmetry in the task completion time of the training group (−4.87 ± 25.12) was significantly lower than that of the control group (29.83 ± 35.48) during the post-training assessment (*t*[30] = −3.14, *p* < 0.001), indicating an increased speed of execution of object transfer with the non-dominant hand chopstick operation that approached the speed of the dominant hand. The planned contrasts revealed no significant differences in the training group post-training assessment (*t*[15] = −0.776, *p* = 0.45) (Figure 4).

### 3.3 Smoothness of joint movement

In a 2 × 2 mixed-design ANOVA of the asymmetry of RMSJ in elbow flexion-extension, with time (pre-training vs post-training) as the within-subjects factor and with a group (training group vs control group) as the between-subjects factor, we found a significant main effect of time (*F*[1,30] = 17.76, *p* < 0.001, partial *η* ^2^ = 0.372) and a significant time × group interaction (*F*[1,30] = 6.95, *p* = 0.01, partial *η* ^2^ = 0.188) (Figure 3). A *post-hoc t*-test showed that the asymmetry of RMSJ in the training group (−1.32 ± 3.76) was significantly lower than that in the control group (2.70 ± 6.02) during the post-training assessment (*t*[30] = −2.26, *p* = 0.03), indicating an improved smoothness in movement of the non-dominant hand for chopstick operation that was close to the smoothness in movement of the dominant hand. The planned contrasts revealed no significant difference in the training group during the post-training assessment (*t*[15] = −1.40, *p* = 0.18) (Figure 4).

In a 2 × 2 mixed-design ANOVA of the asymmetry of RMSJ in shoulder flexion-extension, we found a significant main effect of time (*F*[1,30] = 8.17, *p* < 0.01, partial *η* ^2^ = 0.214) and a significant time × group interaction (*F*[1,30] = 9.60, *p* < 0.01, partial *η* ^2^ = 0.242). A *post-hoc t*-test showed that the asymmetry of the RMSJ in the training group (−0.95 ± 2.37) was significantly lower than that in the control group (1.93 ± 2.89) during post-training assessment (*t*[30] = −3.08, *p* < 0.01), indicating an increased smoothness in movement with the non-dominant hand chopstick operation, bringing it close to that of the dominant hand. The planned contrasts revealed no significant difference in the training group during post-training assessment (*t*[15] = −1.60, *p* = 0.13) (Figure 4).

In a 2 × 2 mixed-design ANOVA of the asymmetry of RMSJ in shoulder abduction-adduction, we observed significant main effects of time (*F*[1,30] = 10.87, *p* < 0.01, partial *η* ^2^ = 0.266) and a significant time × group interaction (*F*[1,30] = 5.59, *p* = 0.03, partial *η* ^2^ = 0.157). A *post-hoc t*-test showed that the asymmetry of RMSJ in the training group (−0.95 ± 3.56) was significantly lower than that in the control group (3.28 ± 5.77) during post-training assessment (*t*[30] = −2.45, *p* = 0.02), indicating an increased smoothness in movement with the non-dominant hand chopstick operation, bringing it close to that of the dominant hand. The planned contrasts revealed no significant difference in the training group during post-training assessment (*t*[15] = −1.07, *p* = 0.30) (Figure 4).

### 3.4 Perceived psychological stress

A 2 × 2 mixed-design ANOVA of the asymmetry of VAS to assess perceived psychological stress, with time (pre-training vs post-training) as the within-subject factor and group (training group vs control group) as the between-subjects factor, revealed a main effect of time (*F*[1,30] = 65.50, *p* < 0.001, partial *η* ^2^ = 0.686) and a significant group × time interaction (*F*[1,30] = 50.83, *p* < 0.001, partial *η* ^2^ = 0.629) (Figure 3). A *post-hoc t*-test showed that the asymmetry of the RMSJ in the training group (0.98 ± 1.04) was significantly lower than that in the control group (2.71 ± 2.32) during the post-training assessment (*t*[30] = −6.29, *p* < 0.001), indicating decreased perceived psychological stress with the non-dominant hand chopstick operation that was close to the perceived psychological stress of the dominant hand. The planned contrasts revealed a significantly higher asymmetry than 0 in the training group during post-training assessment (*t*[15] = 3.81, *p* < 0.01) (Figure 4).

### 3.5 Brain activity

In a 2 × 2 × 2 mixed-design ANOVA of the Oxy-Hb concentration change in the left and right hemispheres with time (pre-training vs post-training) and chopstick use (right hand vs left hand) as the within-subject factors and with a group (training group vs control group) as the between-subjects factor, we found only a significant main effect of chopstick use in the right hemisphere (*F*[1,30] = 4.69, *p* = 0.04, partial *η* ^2^ = 0.135), indicating that the brain activity during left-hand chopstick performance was significantly higher than that during right-hand chopstick performance. However, there was no other significant main effects or interactions (*F* ≤ 2.51, *p* ≥ 0.12 in all cases) (Figure 3).

### 3.6 Relationships among changes in chopstick operation skill, psychological stress, and brain activity

**Figure 5:**
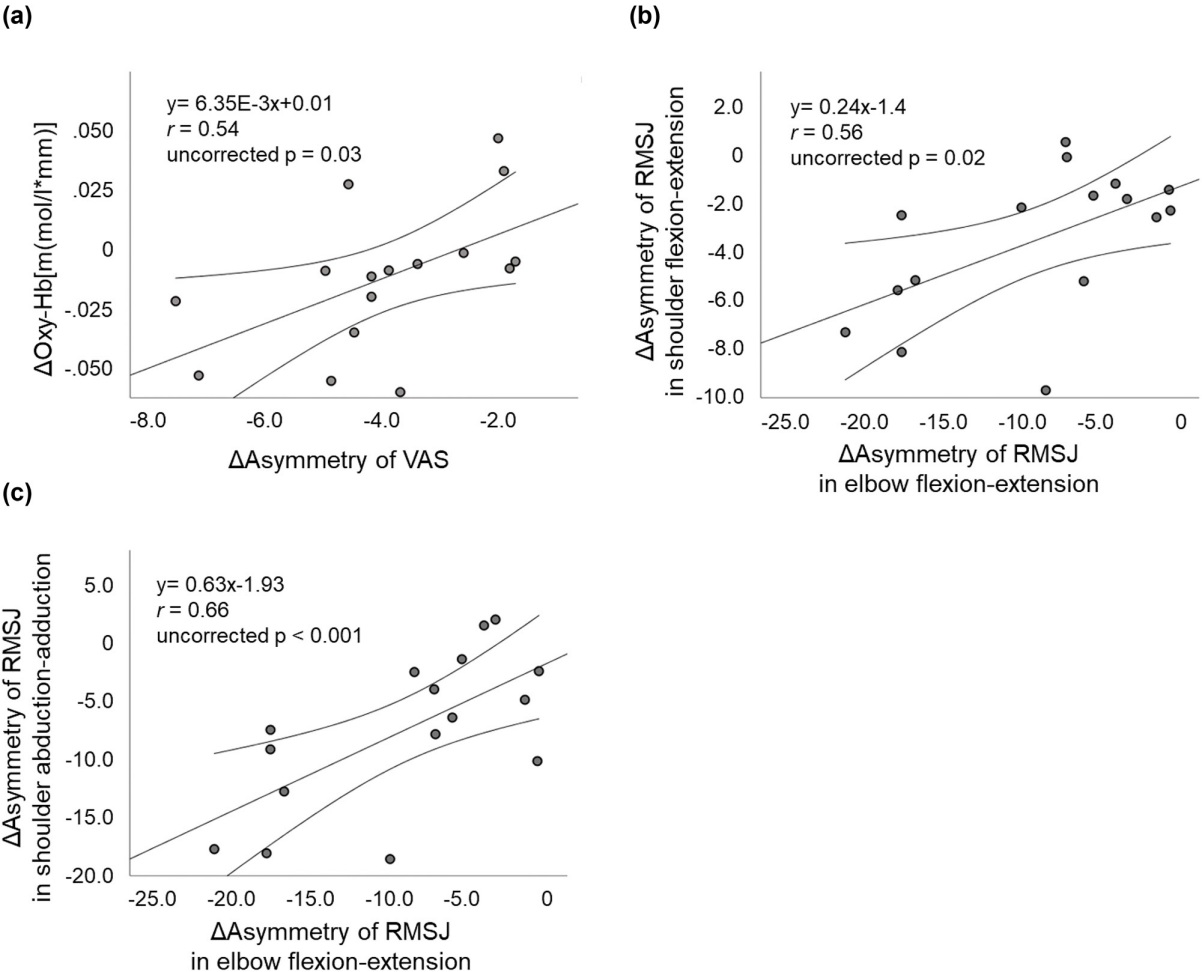
Scatterplots illustrating the relationship between the Δasymmetry of each RMSJ and Δasymmetry of VAS and ΔOxy-Hb concentration in the left hemisphere. (a) A significant positive correlation between Δasymmetry of VAS and ΔOxy-Hb concentration in the left hemisphere. (b) A significant positive correlation between Δasymmetry of RMSJ in elbow flexion-extension and shoulder flexion-extension. (c) A significant positive correlation between Δasymmetry of RMSJ in elbow flexion-extension and shoulder abduction-adduction. The straight and curved lines indicate the mean and 95% confidence interval, respectively.

We found that the change in the asymmetry of VAS was positively correlated with that of the Oxy-Hb concentration in the left hemisphere (*r* = 0.54, uncorrected *p* = 0.03). The change in the asymmetry of RMSJ in elbow flexion-extension was positively correlated with that of shoulder flexion-extension (*r* = 0.56, uncorrected *p* = 0.02) and shoulder abduction-adduction (*r* = 0.66, uncorrected *p* < 0.01) (Figure 5).

## 4 Discussion

A 6-week chopstick training program improved chopstick operation skills, decreased perceived psychological stress, and changed the brain activity. Planned contrasts had no significant differences from the value of zero for mean asymmetry in the marble transferring task and the smoothness of targeted joint movements during the post-training assessment. These results suggest that chopstick operation training of the non-dominant hand improved speed and smoothness of joint movement and nearly reached the performance level of the dominant hand. Moreover, there was no individual joint movement-specific delayed skill acquisition or inadequate patterns. These findings complement other studies with similar findings [8,9,10]. Philip and Frey [9] explained that the improvement in precision drawing of the non-dominant hand occurred in the early phase of learning based on the training-derived functional connectivity changes in the brain. Sawamura et al. [12] suggested that the motor learning phase after 6 weeks of chopstick training was in the early phase of motor learning based on brain activation patterns. Thus, overt measures, smoothness of upper extremity movement, and speed of transferring objects improved earlier than covert measures and psychological stress. These results suggested that chopstick performance of the left-hand approximately reached a level similar to that of the right-hand after 6 weeks of training. However, perceived psychological stress measure, VAS during post-training assessment remained significantly higher. These results also suggested that the performance level of the non-dominant hand may not completely reach that of the dominant hand. However, it has been reported that there are individual differences in perceived psychological stress measured immediately after performing tasks that depend on anxiety and stress levels at the steady state [33,34]. Therefore, the results should be interpreted carefully. Additionally, Shilton et al. [33] have reported that combining measures of autonomic activity (i.e., blood pressure and pulse rate) with anxiety and stress levels at the steady state may be useful to compensate for the validity of the perceived psychological stress immediately after performing a task.

In the untrained dominant hand, speed, smoothness, and perceived psychological stress did not significantly improve between pre- and post-training assessments; thus, there was no inter-limb transfer effect between the non-dominant hand chopstick training in contrast to previous studies [8,9,10]. This indicated that skills more specific to the dominant hand were less susceptible to inter-limb transfer.

We identified the training-derived hemispheric specialization change based on the ROI analysis. We found no significant interaction between Oxy-Hb concentrations in each hemisphere. There was a significantly higher Oxy-Hb concentration in the non-dominant hand than in the dominant hand in the right hemisphere. Previous studies reported that increased ipsilateral hemispheric activity during motor tasks was observed only in the non-dominant hand, while only greater contralateral hemispheric activity was observed in the dominant hand [35,36,37,38,39]. Moreover, this effect is especially apparent during execution of complex movements [35,36]

The changes in asymmetry of chopstick operation skill, psychological stress, and Oxy-Hb concentration revealed positive correlations between changes in the asymmetry of VAS and changes in Oxy-Hb concentration in the left hemisphere and between changes in the asymmetry of RMSJ in elbow flexion-extension and those of shoulder flexion-extension and shoulder abduction-adduction. The close relationships among the RMSJ of all joint movements suggested a tendency toward a level similar to that of the dominant hand. The results also indicated that decreased ipsilateral brain activity in brain regions related to motor learning reflects the decreased psychological stress for a complex task that required a high level of motor performance skill. A large sample study reported a significant positive correlation between asymmetry of motor performance and hand movement differences in ipsilateral deactivation [38]. The results suggested that ipsilateral deactivation was associated with reduced hand lateralization.

This study has some limitations. The execution speed, smoothness of upper extremity joint movement, perceived psychological stress, and brain activity pattern were examined under one condition only. We were unable to determine the extent to which the acquired chopstick operation skill was transferred to various situations during a meal. Moreover, all participants had enough chopstick-use experience with their dominant hands. Thus, the action model of chopstick operation was already stored as memory. Additionally, we set a passive control group that did not experience laboratory training to prove that the change in chopstick operation skills and brain activity deviated from the type of general variations that one would expect from environmental influences. Therefore, we were unable to remove the influence of familiarity with the laboratory equipment and environment due to a laboratory training experience. The fNIRS had a low spatial resolution and could not evaluate the subcortical regions that contributed significantly to motor learning. Finally, this study did not conduct a follow-up assessment post-training. Therefore, we were unable to determine whether the acquired skill was long-lasting.

Varied conditions, follow-up assessments, inclusion of active controls, extrinsic feedback, occupational therapy, and neuromodulation methods are recommended in future studies.

## 5 Conclusion

The 6-week chopstick training program for the non-dominant hand improved speed and smoothness of chopstick operation with that hand and led to the acquisition of a chopstick performance level similar to that of dominant hand chopstick use. These data may be useful in the rehabilitation of patients requiring a change in handedness due to stroke or other conditions affecting the dominant hand. Psychological and functional brain assessments can be helpful for assessing proficiency in chopstick training skills.
